# Automatic extraction of biomolecular interactions: an empirical approach

**DOI:** 10.1186/1471-2105-14-234

**Published:** 2013-07-24

**Authors:** Lifeng Zhang, Daniel Berleant, Jing Ding, Eve Syrkin Wurtele

**Affiliations:** 1 Siemens Corporate Research, Princeton, NJ, USA; 2 Department of Information Science, University of Arkansas at Little Rock, Little Rock, AR, USA; 3 Ohio State University Medical Center, Columbus, OH, USA; 4 Department of Genetics, Cell & Development Biology, Iowa State University, Ames, IA, USA

**Keywords:** Biomolecular interactions, Information extraction, Text mining, Networks

## Abstract

**Background:**

We describe a method for extracting data about how biomolecule pairs interact from texts. This method relies on empirically determined characteristics of sentences. The characteristics are efficient to compute, making this approach to extraction of biomolecular interactions scalable. The results of such interaction mining can support interaction network annotation, question answering, database construction, and other applications.

**Results:**

We constructed a software system to search MEDLINE for sentences likely to describe interactions between given biomolecules. The system extracts a list of the interaction-indicating terms appearing in those sentences, then ranks those terms based on their likelihood of correctly characterizing how the biomolecules interact. The ranking process uses a *tf-idf* (term frequency–inverse document frequency) based technique using empirically derived knowledge about sentences, and was applied to the MEDLINE literature collection. Software was developed as part of the MetNet toolkit (http://www.metnetdb.org).

**Conclusions:**

Specific, efficiently computable characteristics of sentences about biomolecular interactions were analyzed to better understand how to use these characteristics to extract how biomolecules interact.

The text empirics method that was investigated, though arising from a classical tradition, has yet to be fully explored for the task of extracting biomolecular interactions from the literature. The conclusions we reach about the sentence characteristics investigated in this work, as well as the technique itself, could be used by other systems to provide evidence about putative interactions, thus supporting efforts to maximize the ability of hybrid systems to support such tasks as annotating and constructing interaction networks.

## Background

Data mining the biomedical literature, sometimes called the biomedical textome, literaturome, or bibliome, has become increasingly important as the vast amount of textual information now available online promises correspondingly great benefits from automatically processing it. A key category of this information is *interactions*. Comprehensive mining of biomolecular interactions requires determining whether an interaction between entities exists and, if so, what kind of interaction it is. Typically, the interaction is described with an interaction-indicating term (IIT), often a verb.

Automatic extraction from text of information about interactions among biologically relevant entities can target processes such as drug interactions [1,2], transcriptomic interactions, protein-protein interactions (PPIs), and others. To support applications, interaction data extracted by text mining can be stored in biomolecular interaction databases. Such databases are an important enabling technology. For example they facilitate human information seeking and conceptual understanding, and support biomolecular network analysis [3].

A considerable variety of interaction databases have been constructed in recent years. Examples include DIP [4], BioCyc [5], MIPS [6], and MetNet [7], which is the database and toolbox project associated with the present work. Such databases can be based on laboratory research results, like MIPS and KEGG. Alternatively they can be manually curated from biomedical publications, like DIP and BioCyc. While manual curation of existing publications is a quicker way to populate a database than acquiring wet lab results, automatic methods are much quicker still [8]. Thus, researchers have increasingly pursued automatically extracting interactions described in online biomedical texts such as the 22 million-plus records in PubMed.

Methods for automatically extracting interactions from text passages range across a spectrum of complexity from basic co-occurrence analysis, to rule-based template matching, to natural language processing (NLP), including growing interest in shallow methods such as kernel-based approaches. Corpus-based statistical techniques are often used to help leverage automatic extraction methods.

The most basic methods analyze simple co-occurrence of biomolecules within a text unit (e.g. [9,10]). However, much of the information in text is ignored by this approach, implying a tendency to comparatively low precision (but correspondingly higher recall) for detecting interactions compared to more sophisticated approaches that use more of the available information.

Template matching methods may be useful when an interaction template like “A activates B” can be matched to the text [11-13]. Ontologies can be used to match suitably related words together [14]. Syntactic analysis methods parse each text unit and try to match the parsed result with rules (e.g. [15]). These methods can have relatively high precision because of the specific requirements a passage must meet before it is considered a match. However recall tends to be correspondingly lowered because a relevant passage can fail to have the precise word placement characteristics required and thus remain undetected.

Thus, new techniques are needed to improve recall relative to template and closely related methods, while improving precision relative to basic co-occurrence detection alone. Such techniques can, for example, consider frequencies and other corpus-wide features of biomolecules [16,17]. While analysis of individual passages is typically involved, full corpus techniques also use corpus-wide properties, which are inexpressible by methods limited to individual passages. The present work uses this corpus-based strategy.

Ultimately, NLU (computer natural language understanding) will achieve very high levels of both recall and precision to the degree that human language performance levels can be achieved by computers. Thus full natural language understanding (NLU) is the grail of information extraction. NLU is not expected to be feasible for some time, however, syntactically sensitive approaches that do shallow or deep parsing of text can be viewed as steps toward the goal of full NLU, and have gained attention in the PPI literature [18-20]. These approaches increasingly rely on kernel functions ([21-26]). While kernel approaches reduce to feature vector comparisons in theory, they do so in a way that can use implicit rather than explicit features, including features not strongly localized, in particular syntactic dependency relations [27]. Yet such methods continue to require relatively large amounts of computation, making them cumbersome when applied to large corpora. Such issues help motivate investigating a wide variety of other approaches, such as the one described herein.

Crucially, ensembles of techniques used together can perform better than individual techniques used alone [28]. Consequently, it is useful to explore the rich space of possible techniques because they could be usefully combined in hybrid system designs that work better than individual methods, even if some constituent methods seem to perform better than others when tested in isolation on benchmarks.

### From interaction existence to interaction type

Automatic interaction extraction from sentences [29] requires first finding relevant sentences [30]. Given appropriate sentences, an automatic interaction extraction method could focus on determining whether two biomolecules interact [31,32]. Yet *how* they interact, when they do, is also of paramount importance. One approach to this is to classify interactions into predefined categories [33]. Bell et al. [28] extended the interaction category idea to help identify specifics about particular interaction terms, in particular the direction of the interaction, and showed a way to optimize the categorization strategy. The need for even more specific determination of interaction type (e.g. [34]) was a principal motivation for efforts such as the BioNLP’09 [35] and the GENIA Event [36].

The present report addresses a similar problem. As an example, given the pair ‘ATP’ and ‘myosin,’ our method can detect and return that the interaction between them is ‘bind’ or ‘hydrolyze.’ This is a more specific objective than that of our previous report [32], which dealt only with identifying interacting biomolecules, and not with extracting the types of the interactions. Our present method was developed using the MEDLINE corpus, upon which PubMed is based (http://www.nlm.nih.gov/pubs/factsheets/medline.html).

We first examined sentences in biomedical texts and empirically characterized the evidence for interaction provided by efficiently computable sentence traits. Such computationally simple methods can be quite effective in information extraction tasks [37]. More complex and computationally costly sentence characteristics can also be effective [38], but are correspondingly less scalable. Because our method relies on empirically uncovering how passage characteristics provide evidence about biomolecular interactions we refer to the method as *text empirics*.

### Text empirics and machine learning

By text empirics we mean, specifically, the use of statistical properties of text passage characteristics that are efficiently computable for a given passage, and derived by manually mediated analysis of a corpus. Prior to development of the machine learning field this was the only way to determine statistical properties of natural language text. In contrast, machine learning algorithms derive statistical properties more automatically. Machine learning is thus more labor efficient, although presently requiring hand tagging or at least manual feedback in most approaches (e.g. [39-44]). Polajnar et al. [45] describe a method using unlabeled training data. Despite its disadvantage of higher labor cost compared to machine learning (ML), empirical analysis presents some advantages as well. Firstly, ML-derived rules usually include some that, due to incidental statistical flukes in the data, are relatively uninteresting, unlikely to be generally useful, and seem unconvincing in print. Secondly, and perhaps for that reason, publications typically emphasize conclusions about the ML process itself rather than presenting the specific derived rules themselves. Yet specific rules can be readily and directly applied by designers of new systems, because they are disseminated in explicit, human-friendly, usable form, making their identification and dissemination useful.

Empirical text analyses have a classic tradition, including for example work of George Zipf [46,47] and earlier sources he cites. Yet they have been under-represented in the PPI literature, which instead has focused on ad hoc manual identification of rules, templates, etc., and on machine learning-based techniques. A wide range of disparate methods is useful for the field to investigate because multiple methods can be used together to give better results than methods used individually. Thus research contributing to the space of methods such as the present work, remains relevant and important.

## Methods

While our earlier work explored biomolecular pair co-occurrence to extract interactions from single sentences [32], it gave no indication of *the way* they interact. Our present work is designed to extract information about how they interact. Here, we apply a text empirics approach to design an algorithm which extracts which IIT(s) in a given sentence describes the way a given pair of biomolecules in the sentence interact. This single-sentence technique is then extended to combining evidence from multiple sentences found throughout MEDLINE to provide evidence from the experimental literature about how two biomolecules interact. The method starts with finding a list of stems of the IITs tri-occurring in sentences with the biomolecule pair of interest. It concludes by ranking the list of IIT stems based on their probabilities of correctly describing the interaction.

**The challenge**. We consider biomolecular interactions, defined as direct influences (association, regulation, modification, creation, transportation, etc.) between two organic molecules in a living organism. Protein-protein interactions (PPIs) are a prominent example. We used the individual sentence as a unit of analysis [29], and investigated extracting the IITs (interaction-indicating terms) that co-occur with and correctly describe the interaction of a biomolecule pair of interest, while filtering out those IITs that also are present but do not pertain. For example, consider sentences **S1**-**S3** that contain the terms ‘ATP’ and ‘myosin’ (**S1** is a title and titles were treated as sentences).

**S1***. Measurement of the reversibility of****ATP binding****to****myosin****in calcium****-activated****skinned fibers from rabbit skeletal muscle.*[48]

**S2***. A parallel pathway model of****regulation****simulated the effects of Ca(2+) and****ATP****-free****myosin binding****on both equilibrium****binding****of myosin-nucleotide****complexes****to actin and the general features of ATPase activity*[49].

**S3***. In rigor (in the absence of****ATP****, when all the****myosin****heads are rigidly****bound****to the thin filament), a slight decay was observed in the first few microseconds, followed by no****change****in the anisotropy.*[50]

**S1**-**S3 each** contain several IITs. Their canonical forms are: ‘bind,’ ‘activate,’ ‘regulate,’ ‘complex,’ and ‘change.’ ‘Bind’ appears more frequently than the others. On this basis we might hypothesize ‘bind’ as the interaction between ATP and myosin, and manual inspection shows this is indeed true.

Note a distinction between the following cases.

•An IIT is used to describe an interaction in a particular sentence.

•An IIT characterizes an interaction as an independent fact.

One refers to what is said by a given single sentence, while the other refers to a general fact about an interaction. These cases need to be distinguished because of examples like **S3. S3** contains the terms “bound,” “ATP,” and “myosin” and, as other sentences establish, binding is in fact an interaction between ATP and myosin. However **S3** does not describe that interaction because “bound” is used for a different purpose in that sentence.

The probability that a particular IIT describes an interaction of a given biomolecule pair in a given sentence may be determined by a combination of the evidence contributed by different text characteristics of it. Thus, we manually analyzed sentences from the literature to empirically identify useful characteristics that could assist efforts to automatically extract IITs that correctly describe the interactions of given biomolecule pairs.

### Sentence characteristics that suggest a pertinent IIT

To analyze how specific passage characteristics support extracting IITs that describe how a biomolecule pair interacts, the following operational definitions were used.

•*Sentence*. Either an article title, or a word sequence beginning with a capital letter and ending with a period.

•*Phrase.* A word sequence that occurs inside a *Sentence*, and begins and ends with:

•, |; | : | . | **<beginning of the sentence> | <end of a sentence>** |

•**<whitespace>**-**<whitespace>** | ( | )

•where “**|**” means “or.”

•*IIT.* Acronym for “interaction-indicating term*.*” A word that can describe an interaction between two biomolecules, such as ‘activates’ in “A activates B.”

We began by collecting 320 sentences from the results of 10 queries to PubMed. The 10 queries were based on pairs of biomolecules selected by biologist colleagues to represent typical interests. Each sentence was required to contain at least one IIT. The queries were: *nitrite* &*xanthine*, *pyruvate dehydrogenase* &*phosphofructokinase*, *indole acetic acid* &*starch*, *glucose* &*starch*, *glucose-6-p* &*starch*, *carotenoid* &*IPP*, *cre* &*cytokinin*, *acetyl-CoA* &*leucine*, *glucose* &*pyruvate,* and *ATP* &*myosin*.

In this data set there were 770 IIT occurrences, of which 338 correctly described the interaction between the biomolecule pair, as determined by manual inspection and verified by a biologist. For each occurrence of the 770, we manually investigated *IIT syntactic form* as evidence that an IIT correctly describes the interaction of a given biomolecule pair as a general fact (Table 1). Then we investigated *IIT location* similarly. Finally we investigated the effect of *the number of words between IITs and biomolecule names*. Each of these is described, in turn, next.

**Table 1:** Data on likelihoods that interaction-indicating terms (IITs) correctly describe an interaction of the given biomolecule pair, by IIT syntactic form

**IIT form**	**# correct IITs**	**# IITs in corpus**	**%**
Noun	190	353	(54%)
Adj	10	23	(43%)
Adv	0	0	
Verb with -ing ending	42	81	(52%)
Verb with -s or no ending	23	92	(25%)
Past/perfect verb	67	210	(32%)

**Syntactic form.** Table 1 shows how the syntactic forms of IITs relate to the likelihood that they describe how biomolecules interact. The past and perfect verb forms of IITs are sometimes the same, and the frequency of the perfect form is relatively low, so these were lumped together. Noun and present tense forms are also sometimes the same. We did however manually differentiate these, suggesting that using these results in automatic analyses would work best in conjunction with POS tagging to distinguish these forms.

**IIT location.** The present study focuses on extracting information about how biomolecules interact based on the IITs that are textually associated with them*.* We analyzed different configurations of terms within sentences using the following techniques.

1. Compare the case where an IIT is between the two biomolecule names of interest with the case where the IIT is elsewhere in the sentence.

2. Compare the case where the IIT and both biomolecule names tri-occur in the same *phrase* with the case where a phrasal boundary within the sentence intervenes in some way.

These techniques were previously used for the purpose of distinguishing interacting and non-interacting biomolecules [32], and are applied here for the purpose of identifying correct IITs.

Table 2 gives the results of tri-occurrence order across the two cases. As specific examples, here are the two results (1a and 2a) associated with the comparisons (1 and 2) just listed.

1a. If an IIT appeared *between* the two biomolecules, it had a higher probability of correctly describing the interaction than if it was not between (50% vs. 39%). If the IIT is not between the two biomolecule names, it would be either before or after both of them.

2a. If an IIT and a biomolecule pair all occurred together within the *same phrase*, the IIT had a higher probability of correctly describing the interaction between the two biomolecules than if the three terms were not in the same phrase, 50% vs. 37%. If they were not in the same phrase, the IIT could be in a different phrase from the biomolecule names, or it could be in the same phrase as one biomolecule but the other is in a different phrase, or each of the three terms could be in a different phrase.

**Table 2:** Sentence tri-occurrence characteristics

	**All IITs**	**Between biomolecules**	**Not between biomolecules**	**Tri-occurring in a phrase**	**Not tri-occurring in a phrase**
**Correct IITs**	338	164	174	209	129
**Total IITs**	770	327	443	417	353
**% Correct**	44%	50%	39%	50%	37%

Data on phrasal tri-occurrences vs. tri-occurrences which cross phrasal boundaries are shown in columns 5 & 6. Data on interaction-indicating term (IIT) position relative to biomolecule name position are shown in columns 3 & 4.

These results are consistent with an earlier finding that phrasal evidence has higher precision but lower recall than sentential evidence in descriptions of biomolecular interactions [29].

**The effects of distance.** Let the *near distance* be the number of words between an IIT and whichever biomolecule in the pair it is nearest to (or either one if equidistant). The *far distance* is then the number of words between the IIT and the other biomolecule. We investigated the influence of the near and far distances on the likelihood that the IIT correctly describes the interaction.

Some data are shown in Tables 3 and 4. These tables support the intuition that the likelihood that an IIT is correct is higher for closer distances. They also provide the quantitative data needed to determine regression equations.

**Table 3:** Data for likelihood that an interaction-indicating term (IIT) is correct for some representative near distances (see text for details)

**Near distance**	**# correct**	**# in data set**	**% correct**
0	17	19	89%
1	23	33	70%
2	42	63	67%
3	42	65	65%
4	29	78	37%
5	26	80	33%
6	22	54	41%
…	…	…	…
38	0	1	0%

**Table 4:** Data for likelihood that an interaction-indicating term (IIT) is correct, for some representative far distances

**Far distance**	**# correct**	**# in data set**	**% correct**
0	191	302	63%
1	76	168	45%
2	38	99	38%
3	42	106	40%
4	21	73	29%
5	17	57	30%
6	4	38	11%
…	…	…	…
38	0	1	0%

#### The regression equations

The relationship between probability that an IIT is correct and the near or far distance *d* was modeled as:

(1) $$p\left(\mathrm{an}\;\mathrm{IIT}\;\mathrm{is}\;\mathrm{correct}\right)=b_{0}*e^{-b_{1}*d}$$

where the values of parameters *b*_0_ and *b*_1_ are determined from regression analyses on the data synopsized in Tables 3 and 4. Eq. (1) is a nonlinear regression model instead of the more familiar case of linear regression to find a straight line graph, because the data appeared nonlinear. While nonlinear models still more complex than that of eq. (1) are also possible, overfitting becomes an increasing concern as the model gets more complex. We used the JMP software supplied by SAS, which outputs the optimal parameter values given the regression model and the data.

**Figure 1:**
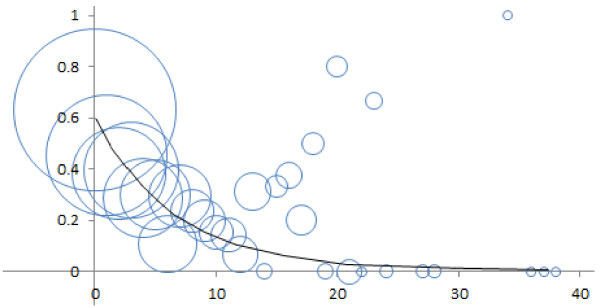
**A nonlinear regression curve for the likelihood that an IIT (interaction-indicating term) is correct (*y *axis) as a function of the *near distance *(*x *axis).** Areas of bubbles are proportional to numbers of sample sentences (near distance is the minimum number of words between an IIT and each biomolecule in the pair).

**Figure 2:**
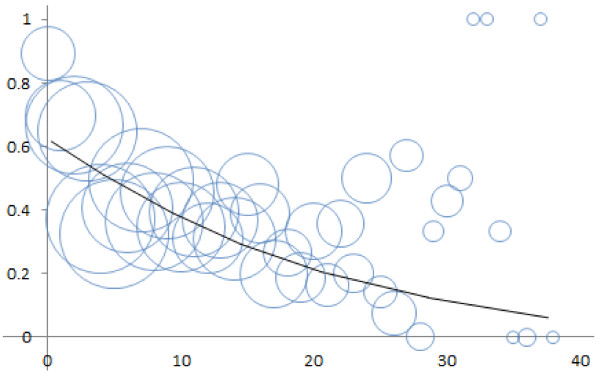
**Regression curve for the likelihood that an IIT (interaction-indicating term) is correct as a function of the *far distance*, which is the maximum number of words between an IIT and each biomolecule name in the pair (cf. Figure**1**).**

The results are shown graphically in Figures 1 and 2. The raw data is represented using bubbles. The area of each bubble is proportional to the number of sample sentences contributing to, and thus adding to the weight of, the data point at the bubble’s center. Each *y*-axis value is the fraction of instances of a given *x*-axis distance in which an IIT correctly characterizes the interaction of that biomolecule pair. A distance of zero means there are zero words between the IIT and a biomolecule. This occurs when the IIT and the biomolecule are adjacent or hyphen-connected. Both cases are illustrated by **S4**.

**S4**. *A rapid equilibration between****myosin-bound ATP****and a myosin-products complex can account for the extra water oxygen incorporation of the product phosphate*[51].

Eq. (2) instantiates the regression curve of eq. (1) for Figure 1 (near distance) and eq. (3) instantiates it for Figure 2 (far distance). Some of the data points in the figures summarize more data than others. In particular, data for longer distances tend to have fewer instances. Thus the data points were weighted by the number of instances they represent. This was to minimize noise distortion in the resulting curves. It also prevents outliers in the figures from unduly affecting the curves.

(2) $$p\left(\mathrm{an}\;\mathrm{IIT}\;\mathrm{is}\;\mathrm{correct}\right)=0.588*e^{-0.14*d}$$

(3) $$p\left(\mathrm{an}\;\mathrm{IIT}\;\mathrm{is}\;\mathrm{correct}\right)=0.605*e^{-0.04*d}$$

### Combining evidence about a sentence

The sentence attributes described above contribute evidence that an IIT describes an interaction between two biomolecules of interest. These sources of evidence may be combined to determine a composite likelihood that the IIT describes an interaction between the biomolecule pair in question. The evidence combination approach we used (eq. 4) is closely related to the naïve Bayes method and is discussed further in Dickerson et al. ([52] section 2.3.3) and Berleant [53]. For additional discussion see Zhang et al. [32], Manning et al. ([54], sections 11.1, 11.3) and Davis ([55], pp. 128–130).

The evidence combination formula is

(4) $$o\left(h\left|f_{1},\ldots ,f_{n}\right)\right)=\frac{o\left(h/f_{1}\right)o\left(h/f_{2}\right)\ldots o\left(h/f_{n}\right)}{o{\left(h\right)}^{n-1}}$$

where *o*(.) refers to odds. Eq. (4) is stated in terms of odds instead of probabilities merely for conciseness. A probability *p* and its corresponding odds are alternative measurements of the same thing and are easily interconvertible: *odds* = *p*/(1-*p*) and *p* = *odds*/(1 + *odds*). Thus in words, eq. (4) expresses the odds of a hypothesis *h* that the IIT in the sentence describes the interaction of the given pair of biomolecules. The formula uses *n* sources of evidence and a default odds *o*(*h*) modeling the entire corpus. The *n* sources, quantified as *o*(*h|f*_*k*_), *k* = 1,…, *n*, each express the odds of *h* given sentence attribute *k*. As applied here, these odds come from the probabilities contributed by the different features discussed earlier. To summarize, these features are:

•Syntactic form of the IIT, with probabilities derived from Table 1.

•IIT location in the sentence, with probabilities similarly derived from Table 2.

•Near distance, with probabilities derived from eq. (2).

•Far distance, with probabilities derived from eq. (3).

### Identifying the interaction between two biomolecules

Applying eq. (4) to each different IIT in a given sentence, we can calculate the chance for each different IIT that it correctly describes the interaction of the biomolecule pair. We used this evidence combination method in an earlier report [32] to investigate *whether* two co-occurring biomolecules interact, and use it here to determine the *way* they interact.

A given sentence containing a pair of biomolecules of interest and IIT(s) can be analyzed to compute the likelihood, for each IIT in the sentence, that it describes how the biomolecule pair interacts. These likelihoods can build up from multiple sentences found in a collection like MEDLINE that provide mutually reinforcing evidence.

For example, consider an IIT stem that tri-occurs with two given biomolecules in the literature more frequently than another IIT stem. The more frequent IIT stem might be conjectured to have a higher probability of correctly describing the interaction of the biomolecule pair. Confounding this, however, is the different background frequencies with which different IITs (and thus their stems) appear in the literature. A commonly appearing IIT stem may tri-occur more frequently in association with a given biomolecule pair than another IIT stem, not because it describes how they interact, but merely because it is a more common IIT overall.

To correct for the varied background frequencies of different IIT stems, we employed the well-known *tf-idf* (term frequency – inverse document frequency) weighting framework.

### Applying the *tf-idf* framework

*Tf-idf* is most familiar as a document retrieval approach (e.g. [54]). It provides a flexible conceptual model readily extended to related problems, such as the present task of identifying the IIT(s) that are descriptive of the interaction between a given biomolecule pair.

We applied the *tf-idf* model by multiplying *tf* and *idf* values; tf*idf then describes the weight of a term *i* as a distinctive characteristic of the document. By using the IIT as the term and modeling the sentences in the corpus that contain the biomolecule pair as the document, we can use this adaptation of *tf-idf* to help identify which IITs are most distinctively associated with the biomolecule pair.

Then, given a pair of biomolecules, we can find all the different IIT stems tri-occurring with the pair, calculate tf*idf for each stem, and return them as a list ranked by the magnitude of tf*idf. IIT stem(s) with higher values of tf*idf are more closely associated with the biomolecule pair, motivating the hypothesis that they are also more likely to correctly describe the interaction. Here are the details of how the *tf-idf* model maps to the present problem. First the *tf* term is discussed, followed by the *idf* term.

#### Calculating term frequency (tf)

In the standard formulation, the term frequency (*tf*) of a term *i* in a document is:

(5) $$tf_{i}=\frac{n_{i}}{\sum_{k}n_{k}}$$

where *n*_*i*_ is the number of occurrences of term *i* in a given document. The denominator thus describes the number of occurrences of all terms in the document and normalizes the *tf* score to be unaffected by document length.

An IIT stem, viewed as a term in (5), might tri-occur unexpectedly frequently in the set of sentences mentioning a given biomolecule pair, where that set is viewed as the document described by (5). This frequency suggests that the IIT stem could describe an interaction of the pair. As a relatively direct measure of the term frequency (*tf*) for the problem here, we used the fraction of those sentences that also contain the IIT stem.

To improve the accuracy of the eq. (5) model, instead of merely counting the sentences, each sentence containing IIT *i* was weighted, and the weights were summed. Weights were based on the likelihood computed from sentence characteristics that the IIT stem correctly describes the interaction of the biomolecule pair. More specifically, weights were calculated using (i) the sentence characteristics described earlier (IIT syntactic form, location, and near and far distances), and (ii) the evidence combination technique of eq. (4). For those sentences that contain multiple instances of the same IIT stem or biomolecule name(s), we used the instance of the IIT stem and of each biomolecule name providing the best likelihood calculation, under the assumption that this satisfactorily estimates the degree to which the sentence constitutes evidence that the biomolecules interact as suggested by the IIT stem.

Accounting for weights in this manner makes the numerator of eq. (5) more complex, but because our objective is to compare different IIT stems tri-occurring with a given biomolecule pair, the denominator does not contribute to the comparison since it is therefore the same for each IIT stem. Therefore the denominator can simply be deleted. At this point, the *tf* calculation of eq. (5) becomes

(6) $$tf_{i}\left(b\right)=\sum_{s}w_{i,s}\left(b\right)$$

where *tf*_*i*_(*b*) is the weight-sensitive term frequency of IIT stem *i* with respect to biomolecule pair co-occurrence *b*, and *w*_*i,s*_(*b*) is the weight of sentence *s* as evidence that IIT stem *i* describes the interaction of pair *b*.

#### Calculating idf

Inverse document frequency (*idf*) measures how well a term separates a small subset of presumably relevant documents from a large subset of presumably irrelevant ones. The traditional formula is

(7) $$\mathrm{id}f_{i}=\log\frac{\left|D\right|}{\left|\left\{d\in D:i\in d\right\}\right|}$$

where |*D*| is the total number of documents in the corpus, and the denominator is the number of documents in which term *i* appears.

A formulation of the inverse document frequency (*idf*) for the present problem that follows naturally from the *tf* discussion above is

(8) $$\mathrm{id}f_{i}=\log\frac{\left|B\right|}{\left|\left\{b\in B:\mathrm{trioccur}\left(i,b\right)\right\}\right|}$$

where trioccur(*i*,*b*) holds if and only if biomolecule pair *b* tri-occurs with IIT stem *i* in at least one sentence in the corpus, where *B* is the set of distinctly different biomolecule pairs co-occurring in sentence(s) in the corpus. Eq. (8) requires finding the number of different biomolecule pairs that an IIT stem appears with, which is tedious because the number of possible different biomolecule pairs is essentially the square of the number of different biomolecules. Therefore to facilitate computation a proxy for eq. (8) was formulated:

(9) $$\mathrm{id}f_{i}\approx \log\frac{\left|\left\{s:j\;\mathrm{in}\;s,\;b\;\mathrm{in}\;s\right\}\right|}{\left|\left\{s:i\;\mathrm{in}\;s,\;b\;\mathrm{in}\;s\right\}\right|}$$

where *s* is a sentence in the corpus, *j* is any IIT stem, *b* is any biomolecule pair, and *i* is a particular given IIT stem. If we have identified all sentences in a comprehensive corpus containing tri-occurrences (the numerator of eq. (9)), and the subset of those sentences containing the IIT of interest (the denominator of eq. (9)), then we can calculate tf*idf from eqs. (6) and (9) to assess the degree to which each IIT stem is characteristic of a given biomolecule pair. Doing the computations based on MEDLINE, a comprehensive corpus, enabled this strategy to more accurately reflect the relationship between IITs and biomolecule pairs as they appear in the biomedical literature. An IIT stem that is highly characteristic of a biomolecule pair then suggests that the IIT stem describes how the pair interacts.

### System development and data collection

**Figure 3:**
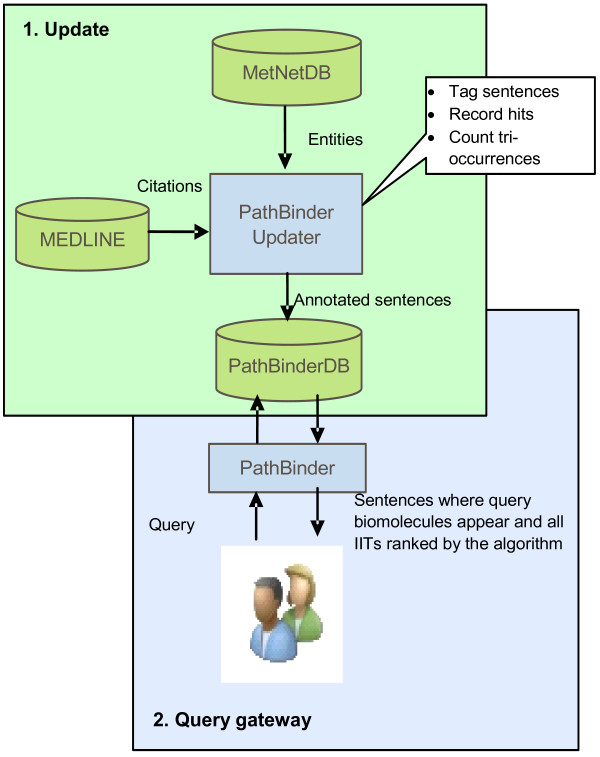
PathBinder system structure, showing a database update module that adds data to the database, and a query module.

To help analyze how well different IIT stems describe the interactions of given biomolecule pairs by using MEDLINE as a source of general facts about biomolecular interactions, we added a major new functionality to the PathBinder software [32]. See Figure 3. PathBinder now applies tf*idf by first querying MetNetDB, the database of the MetNet (*Met*abolic *Net*working, http://www.metnetdb.org[7]) project, to get synonyms associated with biomolecules in the biomolecule pairs of interest. To get a corpus of IITs we used biologists’ suggestions to manually construct and store a lexicon of IIT stems and their inflectional variations. This resulted in 125 IIT stems (App. III of [56]) and 558 distinct IITs.

Next, PathBinder obtained the sentences from MEDLINE that contained tri-occurrences of biomolecule pairs and IITs. The *idf* of each stem was calculated using eq. (9). These sentences were tagged and stored into PathBinder’s own database, PathBinderDB, along with the *idf* for each IIT, for use in calculating the *tf* for an IIT stem given a specified biomolecule pair. PathBinder could then automatically compute the weights of sentences containing the pair, each weight representing the amount of evidence a sentence provided for a particular IIT stem it contained, using eq. (4). This was used in the *tf* calculation of eq. (6).

Thus, PathBinder applies the tf*idf calculation by combining newly determined *tf* values with preprocessed *idf* values. This is how PathBinder merges evidence from the sentences about a given biomolecule pair, calculates a score for each IIT stem appearing in the set of sentences containing the pair, and ultimately ranks the corresponding IIT stem(s) for the pair from best to poorest using their scores.

## Results

More than 30 million sentences in which at least one biomolecule in our lexicon appeared were extracted from MEDLINE and stored in MetNetDB. More than 8 million of these contained at least one tri-occurrence consisting of a biomolecule pair and an IIT. Earlier analysis showed that most biomolecule name co-occurrences take part in tri-occurrences with IIT(s), but less than 22% of tri-occurrences actually describe an interaction. Appendix A in Additional file 1 provides details.

To evaluate our method of ranking the IITs associated with a given biomolecule pair, we randomly chose 200 pairs of biomolecule terms listed in MetNetDB and found by PathBinder to co-occur in sentences of MEDLINE records. Of these, 106 of the biomolecule pairs were both (a) in tri-occurrences, and (b) judged by biologists to actually interact. A test set was then defined, based on: those 106 pairs; all the sentences in MEDLINE in which the pairs co-occurred; and, for each sentence, the different IIT stems it contained, resulting in 1,768 IIT stem occurrences across all the sentences. This test data was used to evaluate how well correct IIT stems could be identified using their rankings. This task was made more challenging by the following factors:

(i) numerous sentences typically exist for a given biomolecule pair,

(ii) many of these sentences contain other biomolecule names as well as multiple IITs, and

(iii) different sentences can describe the same interacting pair using different IIT stems.

For example, one biomolecule pair was ‘chlordecone’ and ‘cytochrome P450.’ Pathbinder returned the ranked list of IIT stems shown in Table 5, of which ‘induc-’ and ‘increas-’ are informative and correct. On the other hand ‘chang-,’ ‘regulat-,’ ‘affect-’ and ‘control-’ are correct, but less informative because the type of interaction is left vague.

**Table 5:** **List of interaction-indicating term (IIT) stems tri-occurring with biomolecule pair chlordecone and cytochrome P450, ranked by *tf-idf *score, i.e., hypothesized likelihood of correctly describing their interaction**

1. induc-	9. (affect-)
2. (chang-)	10. (control-)
3. potentiat-	11. produc-
4. reduc-	12. decreas-
5. (regulat-)	13. bind-
6. increase-	14. lower-
7. (alter-)	15. (metaboliz-)
8. amplif-	16. derive-

Parenthesized IIT stems were classified as vague, thus relatively uninformative.

Vague IITs, though correct, are much less useful for the motivating task of automatically extracting modes of interaction of biomolecule pairs than more specific IITs, because vague affirmations of interaction, like “affect” and “influence,” do not specify the type of interaction. Therefore in addition to analyzing the data for the class of correct IITs (which includes vague ones), we also analyzed the data after removing vague IITs from the lists, leaving lists of *informative* IIT stems for each of the biomolecule pairs in the test set. In both analyses, PathBinder ranked the IIT stems tri-occurring with the pair by *tf-idf* score. Some tri-occurring IIT stems describe the interaction of a nearby biomolecule pair, while others do not. A good ranking strategy will tend to separate these two categories of IITs. Thus from a data set of ranked lists we can test how well rank predicts correctness of an IIT stem. This was our approach to exploring how to distinguish correct IIT stems from incorrect ones.

Although in some cases a pair had only one informative IIT stem in its list, the pair ‘glutathione peroxidase’ and ‘glutathione’ returned 87 correct IIT stems, 74 informative ones and 13 vague ones (Appendix C in Additional file 1). We manually investigated the set of lists of IIT stems and, for each, noted which IIT stem(s) tri-occurring with the associated biomolecule pair correctly characterized the interaction and which did not.

**Figure 4:**
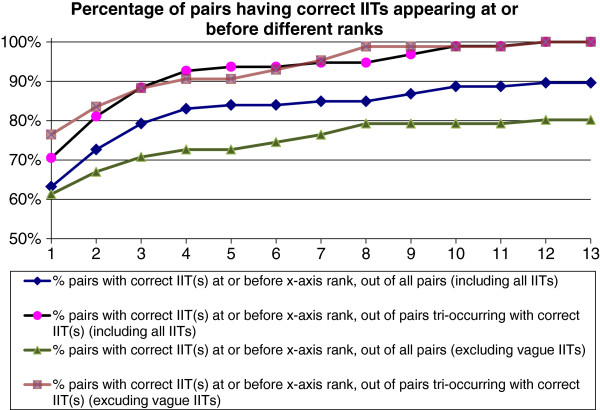
Ability of algorithm to identify at least one correct and informative interaction-indicating term (IIT) stem for a biomolecule pair.

Figure 4 (lower curve) indicates that 80% of the pairs tri-occurred in at least one sentence with a correct and non-vague (i.e. informative) IIT stem from our IIT lexicon, making it potentially possible to automatically identify how the pair interacts. On the other hand, the remaining 20% of the pairs did not. Since vague IIT stems are correct (despite minimal informativeness), including them in the analysis gave an improved curve (shown with diamond-shaped plot points).

The shape of the curve indicates how well the rank determined by the *tf-idf* calculation predicted IIT stem correctness. The curve with square plot points in Figure 4 normalizes the bottom curve, showing the situation for the 80% of the biomolecule pairs for which correct IIT extraction was possible in principle. It shows that in 76% of these a correct IIT stem was ranked first in its associated IIT stem list, in 84% at least one correct IIT stem was ranked first or second, in 88% at least one was in the top-ranked three IIT stems, and in 91% at least one was in the top four. These sub-100% results exemplify an important consequence of applying automatic extraction methods to natural language texts. Since these methods are not at present capable of full understanding of texts, their results cannot be certain, instead providing only some degree of evidence. Highly reliable results thus require a human curation step, until future systems become available that are capable of full NLU, when and if that happens. One possible step in this direction that will be interesting to watch for in the years ahead is the application of IBM’s Watson system or an equivalent to the problem.

We next determined the information retrieval metrics of recall and precision as follows. For each pair of biomolecules, let *N* be the number of IIT stems returned, of which *C* are correct.

•Define the IIT precision *p*(*n*) as the fraction of the top-ranked *n* IIT stems that are in *C.*

•Define the IIT recall *r*(*n*), *n* = 1…*N,* as the fraction of the *C* correct IIT stems that are present in the top-ranked *n* IIT stems.

**Figure 5:**
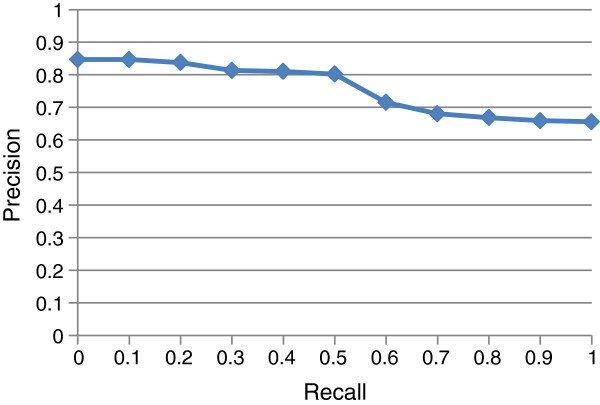
Precision vs. recall of interaction-indicating term (IIT) stems for the test set.

Thus for the various values of *n*, the top ranked *n* informative IIT stems in the IIT stem list of each biomolecule pair have associated IIT recall and precision values *r*(*n*) and *p*(*n*). We computed recall and precision as functions of *n* for each of the test set biomolecule pairs. Figure 5 shows the average precision for different values of recall, using the standard eleven-point interpolated average precision method ([54], p. 146–7). Pairs which tri-occurred only with incorrect IIT stem(s) were excluded since it is impossible to retrieve a correct IIT from a list not containing any.

To place Figure 5 in context, it is based on 231 correct IIT stems out of 1,580 non-vague IIT stems, for a ratio of 0.17 correct IIT stems per incorrect stem, or a precision of just 0.146 for random retrieval. Thus the figure displays significant success in concentrating correct IIT stems into better ranks.

## Discussion

It is instructive to compare the text empirics approach used here with template matching. Existing work focusing on IIT extraction has often used template matching to return a conclusion like “A activates B.” Templates however are inherently restrictive in that some passages will not match any template in a template set. This “falling through the cracks” phenomenon tends to reduce recall.

To help compare template matching and text empirics, it is useful to note the close connection between the two techniques. Whether a passage matches a template is a passage characteristic, and thus can be used as evidence in eq. (4) like other passage characteristics investigated in this report. Thus template sets are well suited to be used as text passage attributes within a text empirics framework. Viewed this way, it is not surprising that some passage characteristics we have considered are somewhat template-like in character. An example is the pattern “a sentence with two biomolecules for which the intervening words contain an IIT.”

Since templates are a subset of the passage characteristics that the text empirics approach can consider, text empirics in general must logically have the potential for higher recall than the template-based approach by itself. Additionally, since an evidence combination strategy like eq. (4) makes incorporating new sources of evidence straightforward, conclusions produced by other techniques can readily be used to improve results.

### A more complex interaction scenario

The discussion so far has not considered cases in which the interaction between two biomolecules as provided by the sentence under consideration is too complex to be described by a single IIT. However, such cases occur. For example consider sentence **S5**.

**S5***.****Glutathione peroxidase****(Se-GPx) is a selenoenzyme which****catalyzes****the****reduction****of hydroperoxides by****glutathione****(GSH), in most mammalian cells.*[57]

The biomolecule pair of interest in this sentence is glutathione peroxidase and glutathione. The interaction between these biomolecules as described here is not named by a single IIT. Instead, the sentence follows the pattern “A catalyzes the reduction of B by C.” Regarding the interaction between A and C, such sentences imply that an interaction exists, but do not describe it explicitly and directly using an IIT.

In the case of sentence **S5**, chemical A (Se-GPx) catalyses a reduction process and C (glutathione) is involved in this process. A trained human can infer that Se-GPx causes oxidation of glutathione, but because the sentence does not actually say this, it would be challenging to design an algorithm to extract the oxidation interaction from the sentence. For our purposes, if we merely want to know whether or not Se-GPx and glutathione interact, this sentence is evidence that they do. But if we want to determine through software what the interaction is then this sentence is likely to mislead the algorithm, because neither of the IIT stems present, ‘catalyz-’ and ‘reduc-,’ describes the interaction of interest, which is oxidation. Therefore, in **S5** we cannot count ‘catalyze’ and ‘reduce’ as correct IITs for the biomolecule pair of interest.

**Figure 6:**
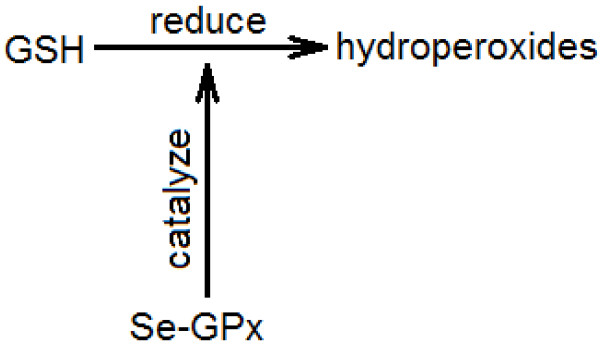
Interaction between a chemical and a process.

We might seek to avoid the “oxidation dilemma” by saying that Se-GPx in **S5** catalyzes, with the affected entity being not a biomolecule but rather a biomolecular process (Figure 6). However, in this work we have aimed at showing how a system could extract a single useful IIT stem describing the interaction between two biomolecules, a model that does not apply in this example.

Alternatively, one might consider sentences such as **S5** as indicating an interaction relation among three biomolecules. Then, when searching for the interaction between A and C the third term B would need to be extracted in addition to the two IITs. This would be harder to do automatically. However it is useful to consider the benefits. There are a number of IITs that sometimes act analogously to ‘catalyze’, such as ‘inhibit’ and ‘stimulate.’ Like other IITs, their stems can appear early in the ranked result lists that are derived and analyzed in the present work, and indeed they can be helpful to biologists as partial characterizations of biomolecular interactions.

## Conclusion

We have described a text empirics approach to mining the biomedical literature for interaction-indicating terms that describe how biomolecule pairs interact. This approach relies on statistical evidence provided by efficiently computable text passage characteristics.

IIT stems that tri-occurred with a given biomolecule pair in a sentence were ranked based on their calculated likelihoods of correctly describing how the biomolecules interact. The precisions of the ranked IIT stem lists returned by the system were at a useful level when the returned lists contained at least one correct IIT. Importantly, while the text empirics approach, like various other techniques, can be applied alone it also has the potential to complement other techniques by being used in conjunction with them. This can improve performance compared to a single technique used alone [28,58], in turn highlighting the importance to the field of investigating the wide space of possible techniques rather than focusing overwhelmingly on finding a single best technique, a task made more challenging in any case because of the difficulty of reliably comparing different PPI extraction methods [26].

As one of many possible ways to combine techniques, template matching and text empirics could be merged in a single system by using empirically derived statistics on the semantics of passages that match a given template compared to the semantics of passages that do not match. Another possibility is to use evidence provided by text empirics to adjust quantitative conclusions about the meanings of passages returned by SVMs on parsed graphs. Alternatively, as in Liu et al. [22], a useful kernel matching function could be defined and then used as a feature, the effectiveness of which would be determined empirically, analogously to the empirical attributes we have discussed in depth in the present work.

The general approach of text empirics we have described could be readily applied in other domains. For example we are currently applying it to extracting neurodevelopmental and ocular development event times from texts. Also the specific empirically determined statistical results described above could be directly applied by others working on protein-protein interaction (PPI) or other biomolecular interaction extraction problems.

## Competing interests

The authors declare that they have no competing interests.

## Authors’ contributions

LZ developed the software and designed and performed the experiments. DB designed the experiments and coordinated the project. JD performed preliminary work and contributed to appendix A in Additional file 1. ESW designed and coordinated integration into MetNet and provided biological expertise. All authors read, approved and contributed to the manuscript .

## Supplementary Material

Additional file 1 Appendices. Click here for file
