# Supplementary material for: Automatic extraction of biomolecular interactions: an empirical approach
Source: BMC Bioinformatics. 2013 Jul 24;14:234. doi: 10.1186/1471-2105-14-234 (PMC3729816; doi:10.1186/1471-2105-14-234)
Supplement: Additional file 1 — Appendices. [file 1471-2105-14-234-S1.pdf]

SUPPLEMENTARY MATERIAL

APPENDICES FOR:

Automatic extraction of biomolecule interaction terms: An empirical approach

Lifeng Zhang[1], Daniel Berleant[2*], Jing Ding [3], and Eve Syrkin Wurtele[4]

[1] Siemens Corporate Research, Princeton, NJ

[2] Department of Information Science, University of Arkansas at Little Rock, Little Rock, AR, 72204

[3] Ohio State University Medical Center, Columbus, OH, 43210

[4] Department of Genetics, Cell & Development Biology, Iowa State University, Ames, IA, 50011

APPENDIX A: PREVALENCE OF USEFUL TRIPLES

We performed a preliminary study to help determine the scope of the problem of extracting, from sentences, triples that describe two interacting biomolecules and an interaction indicating term (IIT) correctly characterizing the interaction. For this we used the IEPA corpus (Appendix B; Ding et. al 2002). Our analysis focused on an enriched subset of triples found in these sentences, namely those for which the IIT was in the same phrase as at least one of the biomolecule names. We called these *admissible sentence triples*. The rationale was that admissible sentence triples would be relatively likely to describe an interaction compared to other triples.

Analysis 1. The first analysis showed that 55% (331 out of 606) of co-occurring biomolecule names associated with one or more admissible sentence triples described an interaction. We concluded the following.

(1) Many co-occurrences did not describe an interaction.

(2) Fewer than 55% of the new admissible sentence triples described an interaction because some biomolecule co-occurrences were due to the presence in the sentence of multiple IITs. Usually, only one of them described an interaction of that biomolecule co-occurrence.

The issue of one co-occurrence being in multiple admissible sentence triples was further investigated in Analysis 2, next.

Analysis 2. Determining what IIT is semantically associated with a particular biomolecule co-occurrence would be easier to get right if every sentence of interest had exactly one co-occurrence and one IIT. However, since many sentences have one co-occurrence and multiple IITs, it is more difficult to determine which is the applicable IIT. Another category of challenging sentences consists of those with *multiple* co-occurrences.

For example, if a sentence contains the three biomolecule names A, B, and C, then it has the three co-occurrences AB, AC, and BC. Matching IITs that may be present with their associated co-occurrences then becomes an issue. To better understand the scope of this problem, we analyzed the same corpus and created Table 6.

The key row in Table 6 is bolded. Note that the restriction to admissible sentence tri-occurrences improves the situation compared to looking at sentence tri-occurrences in general, because the set of admissible sentence tri-occurrences excludes some sentence tri-occurrences that are relatively unlikely to indicate an interaction.

	Total in corpus	# that are part of the description of an interaction	Precision (% that are part of the description of an interaction)	Ratio of tri-occurrence to co-occurrence precisions
Phrase co-occurrences	351	236	0.672365	
Phrase tri-occurrences	839	297	0.353993	0.526489
Sentence co-occurrences	644	334	0.518634	
**Admissible sentence tri-occurrences**	**1833**	**407**	**0.22204**	0.428126
Sentence co-occurrences not also in phrases	293	98	0.334471	
Admissible sentence tri-occurrences not also in phrases	994	110	0.110664	0.330863

Table 6. Comparison of co-occurrences and tri-occurrences, and sentences and phrases, with respect to their richness as sources for mining biomolecular interactions.

Key:

Phrase co-occurrence: two biomolecules present in the same phrase.

Phrase tri-occurrence: an IIT and phrase co-occurrence all within the same phrase.

Sentence co-occurrence: two biomolecules present in the same sentence (a phrase co-occurrence is also a sentence co-occurrence).

Admissible sentence tri-occurrence is defined in the text.

Phrase was operationally defined as follows.

1) The beginning or end of a sentence is also the beginning or end of a phrase.
2) {, ; :} each indicate the end of one phrase and the beginning of the next.
3) <whitespace> – <whitespace> (a dash or hyphen with whitespace on each side) indicates the end of one phrase and the beginning of the next.
4) Left and right parentheses indicate the start and end of a phrase.
5) Only phrases containing a co-occurrence of specific typical biomolecules (analyzed in detail in Deng et al. (2002)), or their synonyms, were considered.

Only 22% of admissible sentence tri-occurrences described an interaction, so that 78% did not. That seemingly challenging fact suggests the possibility of simply ignoring "complicated" sentences and restricting analysis to ones with one co-occurrence and one IIT (that is, one admissible sentence tri-occurrence). Yet doing so might exclude considerable potentially useful data. This issue was investigated in analysis 3, next.

Analysis 3. We investigated the prevalence of multiple IITs in sentences using the same corpus as for *Analysis 1* and *Analysis 2*. Figure 7 shows the results. Even limiting attention to sentences containing just one co-occurrence of a pair of biomolecules (that is, each biomolecule is named once in the sentence), most had more than one IIT (*n* IITs imply *n* tri-occurrences for a sentence with a single co-occurrence). About 10% of the sentences had no IIT, and about 20% had just one, so about 70% had two, three, four, five, and in a few cases even more.

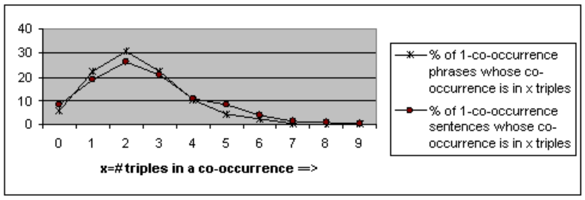

Figure 7. Percentage of co-occurrences associated with 0, 1,… triples, for 1-co-occurrence sentences and phrases.

One may conclude from Figure 7 that restricting attention to sentences with just one IIT would mean ignoring most of the data. But considering sentences with multiple IITs means grappling with the challenge of deciding which IITs properly describe an interaction and which do not, thus motivating the IIT ranking technique presented in this article.

APPENDIX B: The IEPA Corpus

The IEPA corpus may be retrieved from the U.S. National Center for Biotechnology Information at www.ncbi.nlm.nih.gov/entrez/query.fcgi. It consists of the following MEDLINE abstracts in numerical order by PMID:

1645753, 1847862, 1936930, 1940257, 1963324, 2045817, 2163308, 2229283, 2310821, 2328393, 2615438, 3011507, 3070044, 7495469, 7539999, 7581972, 7619502, 7700473, 7743216, 7781763, 7876266, 7884548, 7960031, 7986826, 8105262, 8324162, 8377021, 8527814, 8534418, 8597679, 8629875, 8647319, 8698826, 8713996, 8713999, 8735881, 8735882, 8739893, 8771561, 8777160, 8795088, 8851493, 8853960, 8858923, 8882346, 8886594, 8910395, 8952001, 8987334, 9003017, 9004255, 9005971, 9013600, 9019849, 9042337, 9057099, 9094425, 9096738, 9126376, 9132299, 9135570, 9140228, 9142890, 9159224, 9177227, 9186274, 9187302, 9202317, 9228464, 9231730, 9243706, 9252501, 9268716, 9276734, 9285775, 9291128, 9305858, 9348240, 9350658, 9366557, 9369264, 9374680, 9389497, 9397959, 9442253, 9448714, 9449962, 9459490, 9473570, 9505272, 9507030, 9520493, 9530273, 9561804, 9575789, 9593725, 9605437, 9621246, 9633016, 9634587, 9640660, 9642156, 9642681, 9648822, 9654347, 9660095, 9660200, 9662044, 9666341, 9666695, 9673007, 9682828, 9685679, 9688683,

9689005, 9689469, 9698023, 9722564, 9777024, 9792538, 9792555, 9795377, 9843732, 9844354, 9861026, 9862499, 9863156, 9863156, 9863156, 9867073, 9870709, 9876242, 9877233, 9878732, 9882452, 9892233, 9893131, 9917527, 9920656, 9920735, 9924739, 9924749, 9989288, 9989826, 9990099, 10026815, 10026817, 10066625, 10069063, 10070167, 10071767, 10080713, 10087452, 10094952, 10098482, 10098498, 10102788, 10198105, 10210288, 10235637, 10331406, 10331408, 10334315, 10334320, 10350617, 10364387, 10364415, 10368175, 10375750, 10394640, 10405973, 10422649, 10432317, 10433228, 10441778, 10446397, 10455830, 10469165, 10470379, 10476058, 10477111, 10477529, 10477827, 10480605, 10480887, 10490825, 10492523, 10501197, 10511129, 10516126, 10527125, 10527454, 10545781, 10551836, 10562440, 10563891, 10574951, 10579314, 10600766, 10601847, 10604763, 10610714, 10615945, 10640704, 10652211, 10665337, 10679279, 10679516, 10684630, 10702285, 10702765, 10704947, 10712568, 10728366, 10748000, 10751197, 10757636, 10758829, 10764077, 10767942, 10786701, 10790161, 10795921, 10799298, 10799663, 10805498, 10806392, 10808082, 10810959, 10818263, 10821329, 10822289, 10824737, 10830302, 10837581, 10842667, 10843184, 10844119, 10845452, 10848532, 10852469, 10857385, 10858434, 10858507, 10860673, 10868941, 10868941, 10868951, 10868965, 10874727, 10875926, 10878420, 10879675, 10880376, 10898738, 10908568, 10909425, 10909965, 10913438, 10921449, 10923281, 10923627, 10930542, 10944430, 10947866, 10952979, 10958819, 10964976, 10967095, 10967302, 10969838, 10969914, 10971807, 10988071, 10997289, 10997599, 10997599, 10997600, 10997614, 10997615, 10997617, 10997621, 10997641, 10997646, 10997654, 11001766, 11016897, 11021637, 11024558, 11024559, 11024560, 11024567, 11027362, 11033311, 11042115, 11053526, 11054597, 11062072, 11068095, 11069123, 11082535, 11094982, 11108730.

APPENDIX C: The IIT Stems Associated with Pair Glutathione Peroxidase and Glutathione

The 74 informative IIT stems are listed in order of rank from best to least good.

reduc-
deplet-
oxidat-
decreas-
increase-
reduc-
detoxif-
produc-
synthesiz-
inhibit-
conjugat-
lower-
overexpress-
enhance-
catalys-
induc-
oxidiz-
catalyz-
elevat-
generat-
bind-
form-
transform-
combin-
associate-
maintain-
stimulat-
limit-
attenuate-
augment-
accompany-
activat-
accumulate-
link-
rais-
decarboxylat-
downregulat-
disrupt-
diminish-
modulat-
derive-
modif-
reconstruct-
suppress-
coupl-
incorporate-
remov-
encod-
eliminate-
improve-
distribut-
depress-
stabiliz-
block-
degradat-
upregulat-
complex-
ris-
potentiat-
antagoniz-
agoniz-
coexpress-
releas-
carboxylat-
nitrat-

counteract-
receiv-
blunt-
promot-
hydrolyze-
destabilize-
obliterate-
transport-
diminut-
